# Multiple E3 ligases act as antiviral factors against SARS-CoV-2 via inducing the ubiquitination and degradation of ORF9b

**DOI:** 10.1128/jvi.01624-23

**Published:** 2024-05-06

**Authors:** Miao Yu, Jie Li, Wenying Gao, Zhaolong Li, Wenyan Zhang

**Affiliations:** 1 Department of Infectious Diseases, Infectious Diseases and Pathogen Biology Center, Key Laboratory of Organ Regeneration and Transplantation of The Ministry of Education, The First Hospital of Jilin University, Changchun, Jilin, China; 2 Department of Geriatrics and Special medical treatment, The First Hospital of Jilin University, Changchun, Jilin, China; 3 Institute of Virology and AIDS Research, The First Hospital of Jilin University, Changchun, Jilin, China; The Peter Doherty Institute for Infection and Immunity, Melbourne, Australia

**Keywords:** SARS-CoV-2 ORF9b, HUWE1, UBR4, UBR5, degradation

## Abstract

Severe acute respiratory syndrome-coronavirus-2 (SARS-CoV-2) open reading frame 9b (ORF9b) antagonizes the antiviral type I and III interferon (IFN) responses and is ubiquitinated and degraded via the ubiquitin-proteasome pathway. However, E3 ubiquitin ligases that mediate the polyubiquitination and degradation of ORF9b remain unknown. In this study, we identified 14 E3 ligases that specifically bind to SARS-CoV-2 ORF9b. Specifically, three E3 ligases, HECT, UBA, and WWE domain-containing E3 ubiquitin protein ligase 1 (HUWE1), ubiquitin protein ligase E3 component n-recognin 4 (UBR4), and UBR5, induced K48-linked polyubiquitination and degradation of ORF9b, thereby attenuating ORF9b-mediated inhibition of the IFN response and SARS-CoV-2 replication. Moreover, each E3 ligase performed this function independent of the other two E3 ligases. Therefore, the three E3 ligases identified in this study as anti-SARS-CoV-2 host factors provide novel molecular insight into the virus-host interaction.

**IMPORTANCE:**

Ubiquitination is an important post-translational modification that regulates multiple biological processes, including viral replication. Identification of E3 ubiquitin ligases that target viral proteins for degradation can provide novel targets for antagonizing viral infections. Here, we identified multiple E3 ligases, including HECT, UBA, and WWE domain-containing E3 ubiquitin protein ligase 1 (HUWE1), ubiquitin protein ligase E3 component n-recognin 4 (UBR4), and UBR5, that ubiquitinated and induced the degradation of severe acute respiratory syndrome coronavirus 2 (SARS-CoV-2) open reading frame 9b (ORF9b), an interferon (IFN) antagonist, thereby enhancing IFN production and attenuating SARS-CoV-2 replication. Our study provides new possibilities for drug development targeting the interaction between E3 ligases and ORF9b.

## INTRODUCTION

Severe acute respiratory syndrome coronavirus 2 (SARS-CoV-2) genome consists of a single-stranded positive-sense RNA of approximately 29,900 nucleotides arranged into 14 open reading frames (ORFs), including two replicative polyproteins (pp1a and pp1ab) that are cleaved into 16 non-structural proteins, 4 structural proteins, and 11 accessory proteins (ORF3a, ORF3b, ORF3c, ORF3d, ORF6, ORF7a, ORF7b, ORF8, ORF9b, ORF9c, and ORF10) (1). Although extensive efforts have been made, more in-depth and systematic understanding of SARS-CoV-2 pathogenesis is necessary to prevent and control viral transmission (2).

ORF9b is a 97 aa long protein encoded by an alternative ORF within the nucleocapsid (*N*) gene (3, 4). SARS-CoV-2 infection results in the immediate accumulation of ORF9b, which acts as an antagonist of antiviral type I and III interferon (IFN) responses by targeting multiple components of the innate antiviral signaling pathways (5, 6). Alpha variant markedly increases the subgenomic RNA and protein levels of ORF9b, and ORF9b expression alone inhibits the innate immune response via interaction with the translocase of outer mitochondrial membrane 70 (TOM70), a mitochondrial protein required for the activation of mitochondrial antiviral-signaling protein (MAVS) (7). Therefore, ORF9b is an important drug target to attenuate the virulence of SARS-CoV-2. We recently reported that ORF9b is ubiquitinated and degraded by some E3 ligases, whereas the deubiquitinase (DUB), ubiquitin-specific peptidase (USP29), promotes the virulence of SARS-CoV-2 by preventing ORF9b degradation (8). However, the E3 ubiquitin ligases that mediate the ubiquitination of ORF9b remain unknown.

Protein ubiquitination is a stepwise process carried out by ubiquitin-activating enzymes (E1), ubiquitin-conjugating enzymes (E2), and ubiquitin ligases (E3) (9, 10). Notably, E3 ubiquitin ligases determine substrate specificity, thereby forming a crucial part of the ubiquitination cascade (11). Humans express more than 600 E3 ligases, which are classified into three major categories depending on their enzymatic domain structure and mechanism of action: really interesting new gene (RING) type, homologous with E6-associated protein C-terminus (HECT) type, and RING-between-RING type (12). HECT E3 ligases are characterized by a conserved C-terminal HECT domain (approximately 350 amino acids; approximately 40 kDa) containing a catalytic cysteine that participates in the transfer of ubiquitin to the substrate (13). HUWE1 is a 482 kDa HECT, ubiquitin-associated domain (UBA), and WWE domain-containing E3 ligase that targets several substrates for polyubiquitination and is implicated in inflammatory diseases, tumorigenesis, regulation of cell proliferation, and apoptosis (12, 14). Humans encode seven N-end rule pathway E3 ligases, ubiquitin protein ligase E3 component n-recognin (UBR1–7), four of which (UBR1, UBR2, UBR4, and UBR5) contain an N-degron recognition UBR box (15). UBR5 (309  kDa) is the only protein that belongs to the HECT family (16). UBR5 assembles the K11- to K48-linked branched ubiquitin chains and has various substrates that function as key regulators of the ubiquitin-proteasome system (UPS) in cancer and developmental biology (17, 18). UBR4 remains insufficiently characterized because of the difficulty in manipulating this gene due to its high molecular weight (574 kDa) (19). UBR4 selectively targets substrates for proteasomal degradation (20, 21).

In this study, we identified three E3 ubiquitin ligases, HUWE1, UBR4, and UBR5, that specifically interact with and induce the ubiquitination and degradation of SARS-CoV-2 ORF9b, thereby attenuating ORF9b-mediated IFN inhibition and SARS-CoV-2 replication as antiviral factors. Our findings reveal a new mechanism by which the host can antagonize SARS-CoV-2 and provide novel strategy for drug development against SARS-CoV-2.

## RESULTS

### Multiple E3 ubiquitin ligases specifically interact with and regulate SARS-CoV-2 ORF9b stability

**Fig 1:**
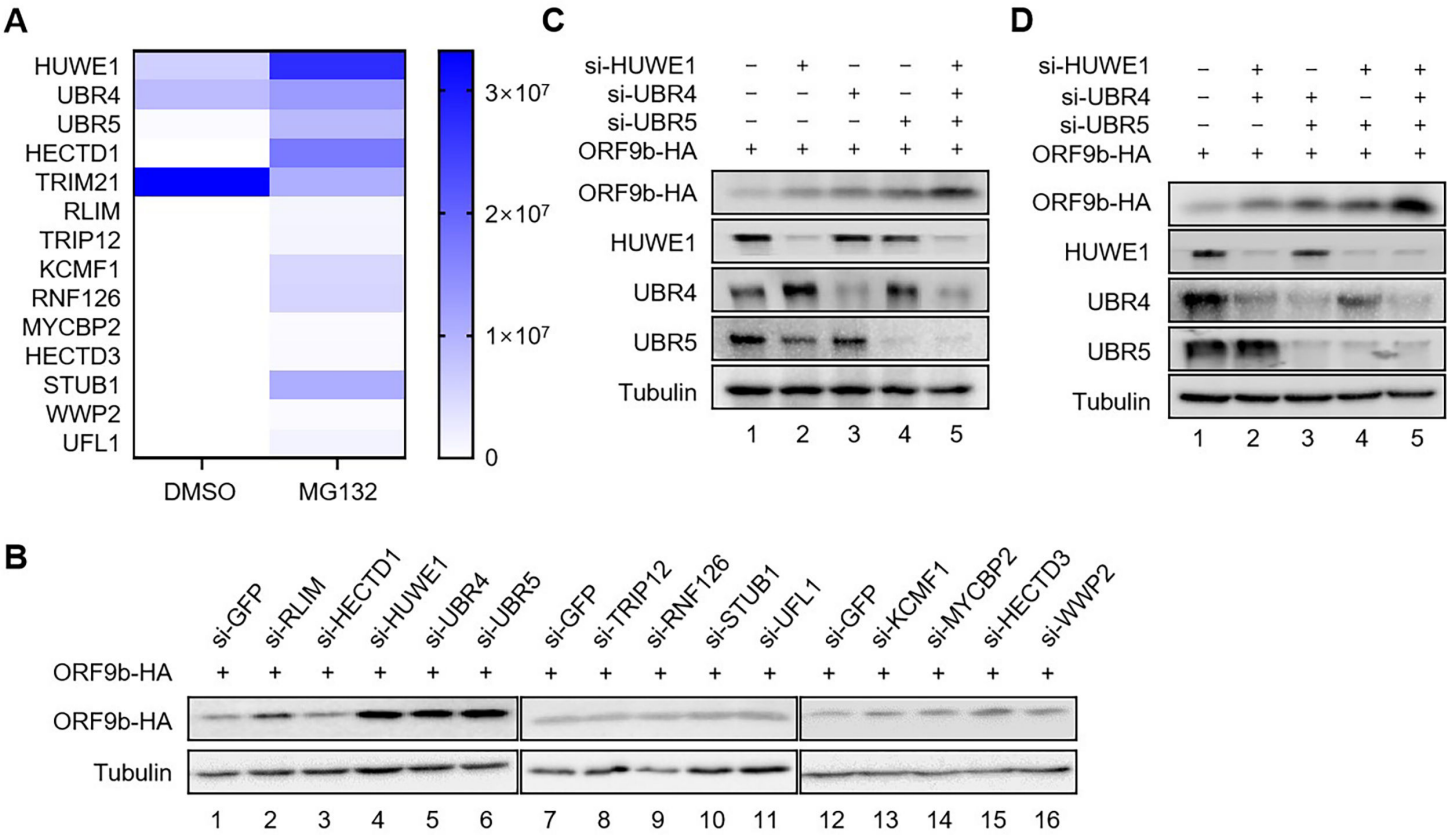
Multiple E3 ubiquitin ligases specifically interact and regulate SARS-CoV-2 ORF9b stability. (**A**) A heatmap of 14 E3 ubiquitin ligases was plotted based on the interacting proteins of SARS-CoV-2 ORF9b as quantified by MS analysis. (**B**) Identification of the E3 ligases HUWE1, UBR4, and UBR5 mediated ORF9b degradation. Each siRNA of E3 ligases interacting ORF9b or siRNA targeting green fluorescent protein (GFP) as negative control plus the ORF9b-hemagglutinin (ORF9b-HA) expression vector were transfected into HEK293T cells for 48 h. Cells were harvested and analyzed with IB of anti-HA and anti-tubulin antibodies. (**C and D**) Cells were transfected with siRNA negative control, HUWE1 siRNA, UBR4 siRNA, UBR5 siRNA, or combinations of siRNA plus ORF9b-HA for 48 h. Cells were harvested and the knockdown of the E3 ligases and the protein level of ORF9b were confirmed by IB with the indicated antibodies.

We recently showed that the stability of SARS-CoV-2 ORF9b is regulated by the ubiquitin-proteasome pathway and that DUB USP29 deubiquitinates its polyubiquitination and reverses its degradation (8). However, the E3 ubiquitin ligases that mediate polyubiquitination of ORF9b have not yet been elucidated. To screen E3 ubiquitin ligases that bind to ORF9b, we co-immunoprecipitated ORF9b transfected HEK293T cells treated with the proteasome inhibitor MG132, prior to harvest to avoid ORF9b degradation, followed by mass spectrometry (MS) analysis (Fig. 1A). We identified 14 E3 ligases that specifically interacted with ORF9b. To investigate their effects on the stability of ORF9b, these E3 ligases were knocked down using small interfering RNAs (siRNAs). Immunoblotting (IB) analysis revealed that the knockdown of HUWE1, UBR4, or UBR5 significantly enhanced the ORF9b stability compared to the knockdown of other E3 ligases and negative control siRNA (Fig. 1B). The regulatory effects of these three E3 ligases on ORF9b stability were further verified using siRNAs (Fig. 1C). Compared to the knockdown of individual E3 ligases, simultaneous knockdown of HUWE1, UBR4, and UBR5 significantly increased the ORF9b stability (Fig. 1C).

To determine whether the three E3 ligase-mediated degradations of ORF9b are independent of each other or act in concert, we simultaneously silenced two or three ligases as indicated. Knockdown of two of three E3 ligases could not rescue the stability of ORF9b as that with knockdown of three E3 ligases (Fig. 1D, lane 5), suggesting that any of the E3 ligases alone can induce ORF9b degradation.

### HECT domain is necessary for HUWE1- and UBR5-induced proteasome-dependent degradation of ORF9b

**Fig 2:**
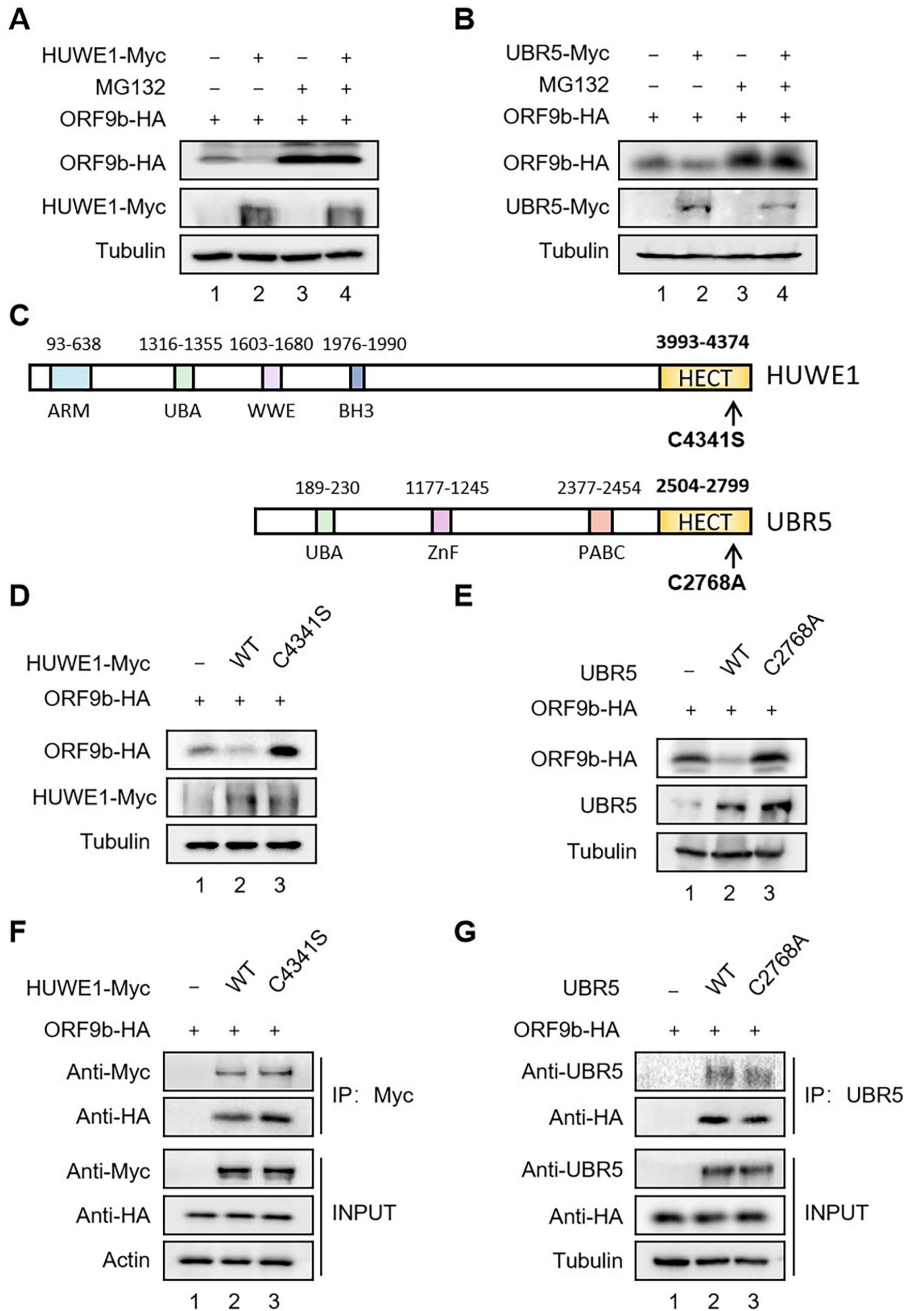
HUWE1 and UBR5 induce the degradation of ORF9b in an enzyme activity-dependent manner. HEK293T cells were transfected with ORF9b-hemagglutinin (ORF9b-HA) and empty vector or HUWE1 wild-type (WT)-Myc (**A**) or UBR5 WT-Myc plasmid (**B**) for 48 h, and treated with or without 10 µM MG132 for 12 h prior to harvest and analyzed by IB with the indicated antibodies. (**C**) Schematic representation of HUWE1 and UBR5 protein structure and their mutants. The domain abbreviations used are as follows: ARM, armadillo repeat-containing domain; BH3, Bcl-2 homology 3 domain; HECT, homologous to the E6AP C-terminus; PABC, polyadenylate-binding protein C-terminal domain; UBA, ubiquitin-associated domain; WWE, Trp-Trp-Glu domain; ZnF, Zinc finger domain. (**D**) HEK293T cells were transfected with ORF9b-HA and empty vector or HUWE1 WT-Myc or HUWE1 C4341S-Myc mutant for 48 h. IB was performed with anti-HA, anti-Myc, and anti-tubulin. (**E**) HEK293T cells were transfected with ORF9b-HA and empty vector or UBR5 WT or UBR5 C2768A mutant for 48 h. ORF9b and UBR5 were analyzed with IB of anti-HA or anti-UBR5 or anti-Tubulin antibodies. (**F**) The interaction of ORF9b and HUWE1 WT-Myc or C4341S-Myc mutant. HUWE1 WT-Myc or C4341S-Myc mutant were co-transfected with ORF9b-HA into HEK293T cells. Cells were treated with 10 µM MG132 for 12 h prior to harvest. HUWE1 was immunoprecipitated with Myc antibody, and the associated ORF9b was detected with HA antibody. (**G**) The interaction of ORF9b and UBR5 WT or C2768A mutant. UBR5 WT or C2768A mutant were co-transfected with ORF9b-HA into HEK293T cells. Cells were treated with 10 µM MG132 for 12 h prior to harvest. UBR5 was immunoprecipitated with endogenous UBR5 antibody, and the associated ORF9b was detected with HA antibody.

To verify the effects of the three E3 ligases on ORF9b stability, we examined whether the overexpression of individual E3 ligases induces ORF9b degradation in the presence or absence of the proteasome inhibitor MG132 in HEK293T cells. We only successfully constructed HUWE1- and UBR5-expressing vectors because of the high molecular weight of UBR4, resulting in difficult manipulation. Overexpression of both HUWE1 and UBR5 significantly reduced the stability of ORF9b, whereas MG132 treatment reversed this effect (Fig. 2A and B), confirming that HUWE1 and UBR5 induce ORF9b degradation via a proteasome-dependent pathway.

HUWE1 and UBR5 belong to the HECT E3s family (22). Each HECT-type E3 contains a characteristic HECT domain at its C-terminus, which catalyzes the transfer of ubiquitin from E2 to itself and then to a specific substrate (23). To confirm whether the HECT domains of HUWE1 and UBR5 are required for ORF9b degradation, we generated two HECT domain mutants, HUWE1 C4341S and UBR5 C2768A, respectively (Fig. 2C) (11, 22). Overexpression of HUWE1 or UBR5 mutant did not lead to the degradation of ORF9b compared to wild-type (WT) HUWE1 or UBR5 (Fig. 2D and E). Moreover, co-immunoprecipitation (co-IP) analysis showed that the two mutants maintained their binding ability with ORF9b (Fig. 2F and G).

### Knockdown of HUWE1, UBR4, or UBR5 reduces the degree of ORF9b ubiquitination

**Fig 3:**
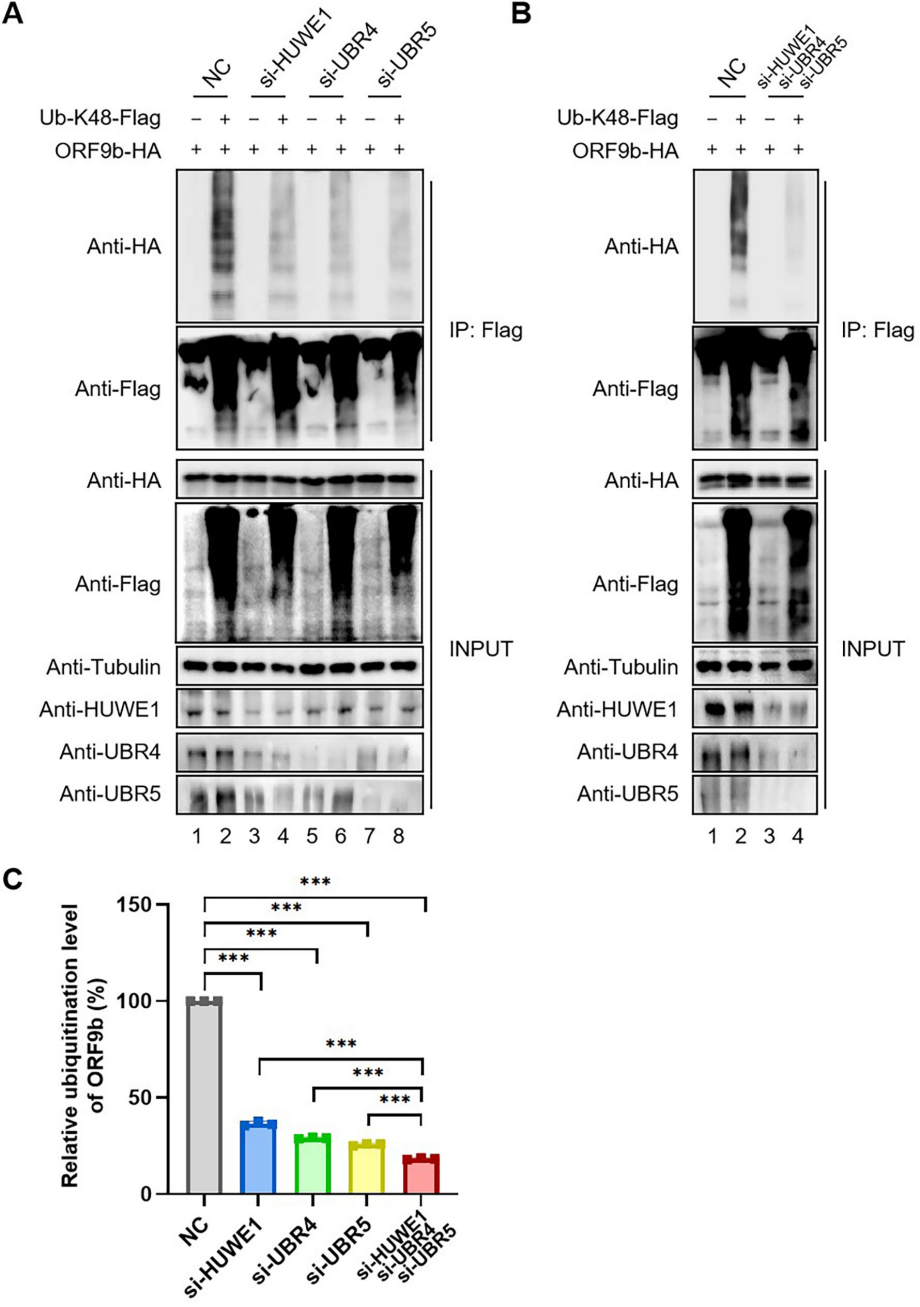
siHUWE1, siUBR4, or siUBR5 alone or combination all reduced the polyubiquitination of ORF9b. (**A and B**) Negative control (NC) siRNA, siHUWE1, siUBR4, siUBR5, or combination of three siRNAs were co-transfected with ORF9b-hemagglutinin (HA) plus K48-only ubiquitin-Flag (Ub-K48-Flag) into HEK293T cells. Lysates of cells were subjected to Flag IP, and then analyzed by IB. (**C**) Quantification of ORF9b ubiquitination assay results was shown in (**B**). The intensity of the Ub band in IP lanes was quantified using ImageJ software and the siNC group was set to 100%. All data are representative of three independent experiments. The data are presented as means ± SD (***, *P* < 0.001).

Polyubiquitination occurs at one of the seven lysine residues in ubiquitin, resulting in the formation of different types of ubiquitin chains that dictate the fate of the modified protein (24, 25). To investigate the effect of HUWE1, UBR4, or UBR5 knockdown on ORF9b polyubiquitination, we selected the K48-only ubiquitin-expressing vector, Ub-K48-Flag, to perform a co-IP assay as ORF9b is ubiquitinated and degraded via a K48-linked modification (8). Knockdown efficiencies of the three E3 ligases were confirmed via IB analysis in HEK293T cells (Fig. 3A). Co-IP assays showed that HUWE1, UBR4, and UBR5 knockdown all reduced the polyubiquitination of ORF9b (Fig. 3A). Furthermore, simultaneous knockdown of the three E3 ligases resulted in a more profound reduction in ORF9b polyubiquitination than the knockdown of individual E3 ligases (Fig. 3B and C).

### ORF9b K4 and K40 are ubiquitinated sites of E3 ligases HUWE1, UBR4, and UBR5

**Fig 4:**
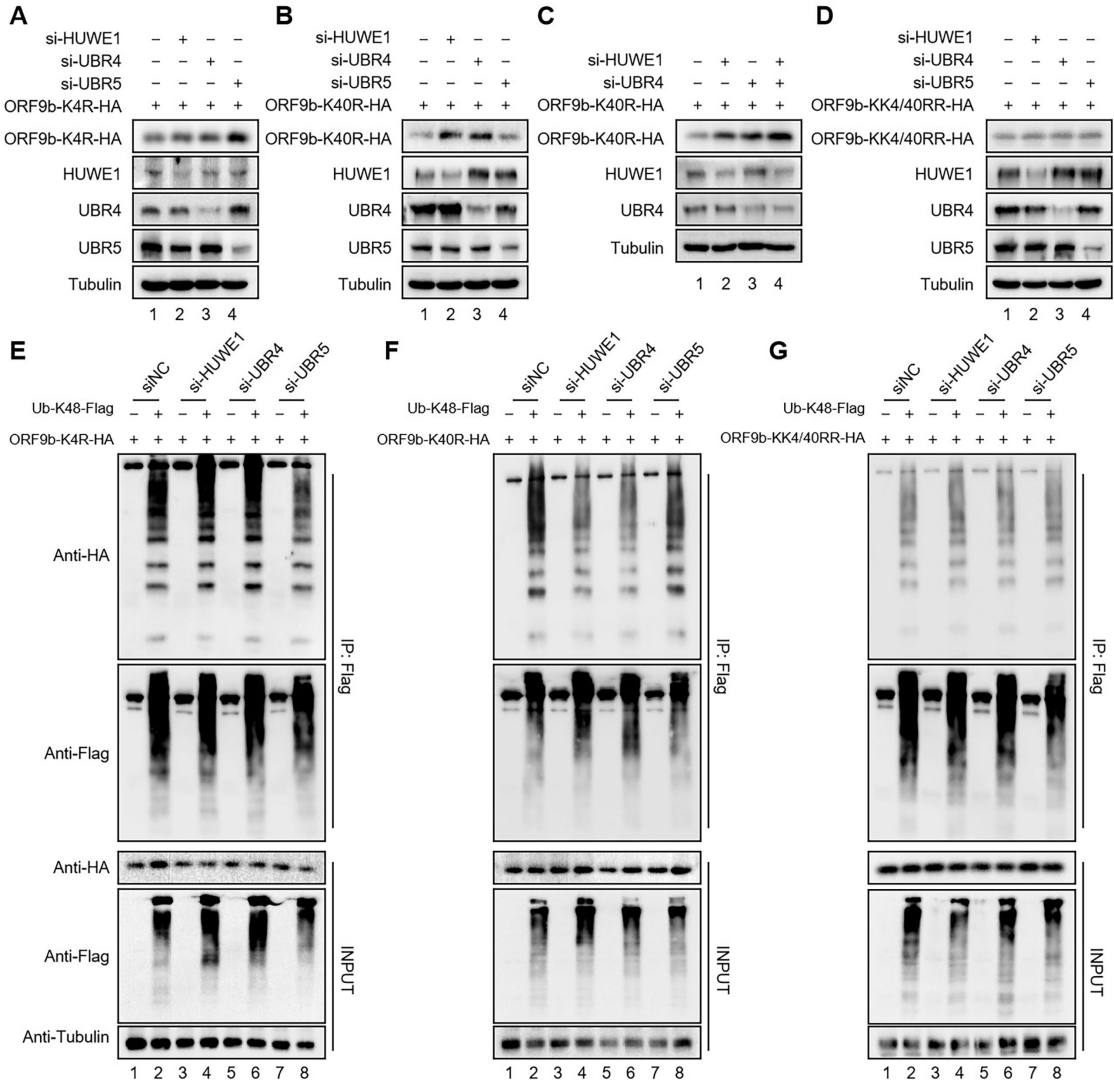
Lysine 4 in ORF9b is ubiquitination site for HUWE1 and UBR4, and lysine 40 is for UBR5. (A–D) HEK293T cells were transfected with negative control (NC) siRNA or HUWE1 siRNA or UBR4 siRNA or UBR5 siRNA plus ORF9b-K4R, K40R, or KK4/40RR mutant as indicated. Knockdown of E3 ligases and the protein level of ORF9b were confirmed by IB with the indicated antibodies. (E–G) HEK293T cells were transfected with NC siRNA, siHUWE1, siUBR4, or siUBR5 plus ORF9b-hemagglutinin (HA) mutant and Ub-K48-Flag as indicated. Lysates of cells were subjected to Flag IP and analyzed by IB.

Lysines 4 and 40 in ORF9b are the ubiquitination sites of E3 ligases (8). To determine the exact site of ORF9b that is ubiquitinated by HUWE1, UBR4, or UBR5, we examined the effects of HUWE1, UBR4, and UBR5 on the stability of ORF9b WT or mutants harboring individual K4R, K40R, or combined KK4/40RR in HEK293T cells. HUWE1 or UBR4 knockdown had no effect, whereas UBR5 knockdown increased the ORF9b-K4R stability, indicating that K4 may be the ubiquitination site of HUWE1 and UBR4, but not UBR5 (Fig. 4A). In contrast, the stability of the ORF9b-K40R mutant was regulated by HUWE1 or UBR4 knockdown but not by UBR5 knockdown, indicating that K40 is the ubiquitination site of UBR5 (Fig. 4B). Moreover, the stability of ORF9b-K40R mutant was higher in HUWE1 and UBR4 double-knockdown cells than HUWE1 or UBR4 single-knockdown cells (Fig. 4C). Therefore, it was presumed that HUWE1 and UBR4 independently ubiquitinate the K4 site of ORF9b.

To further confirm this hypothesis, we examined whether HUWE1, UBR4, or UBR5 affected the ubiquitination level of ORF9b mutants using a co-IP assay. Interestingly, HUWE1 or UBR4 knockdown had no influence on the ubiquitination level of ORF9b-K4R, and UBR5 knockdown had no effect on the ubiquitination level of ORF9b-K40R (Fig. 4E and F). Furthermore, knockdown of the E3 ligases had no effect on the stability or ubiquitination level of KK4/40RR, further indicating that K4 and K40 are the ubiquitination sites of different E3 ligases (Fig. 4D and G). Our findings suggest that ORF9b-K4 is the ubiquitination site of HUWE1 and UBR4, whereas K40 is the ubiquitination site of UBR5.

### HUWE1, UBR4, and UBR5 affect the ORF9b-mediated inhibition of IFN response

**Fig 5:**
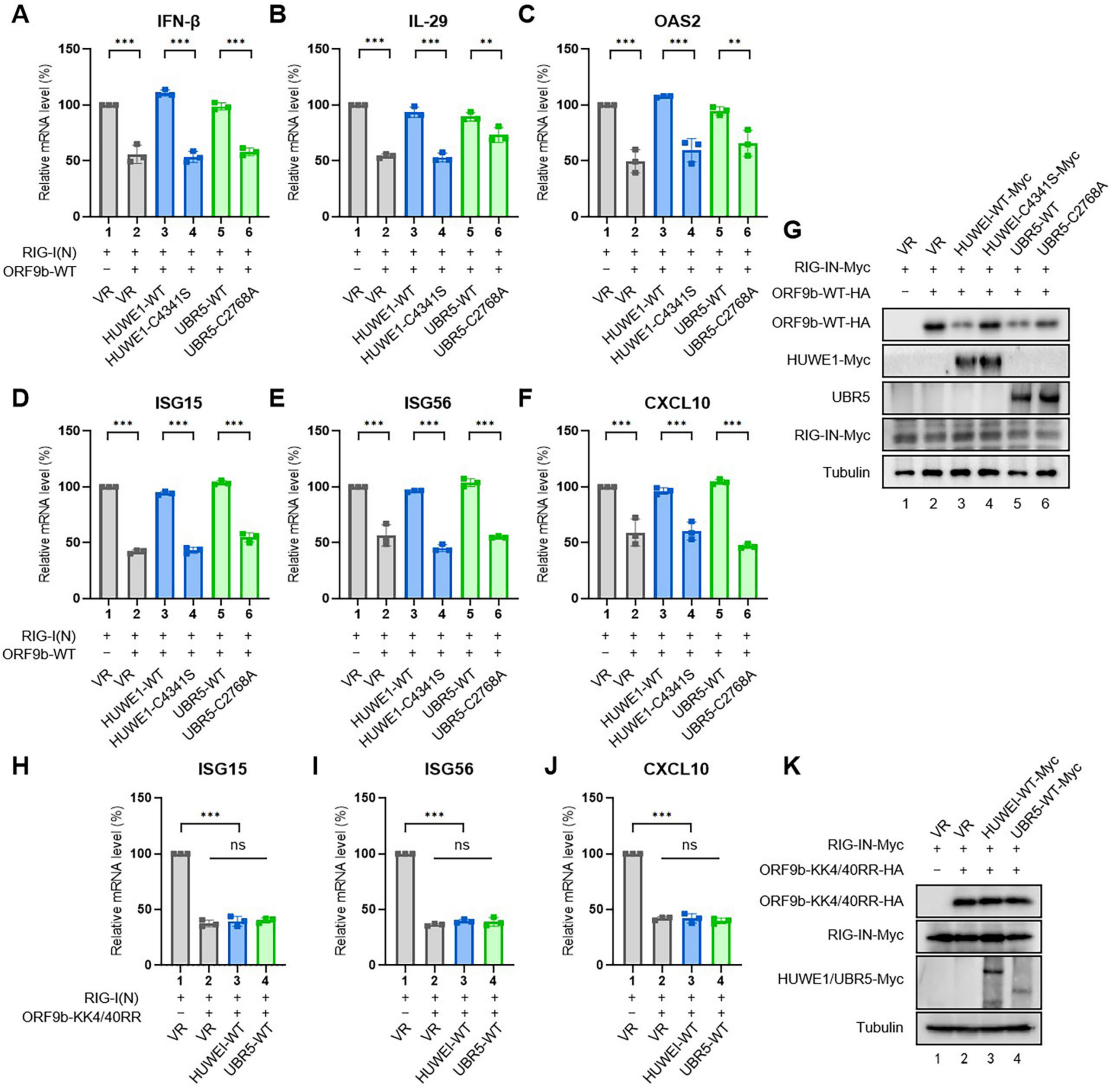
HUWE1 and UBR5 attenuate ORF9b-mediated IFN inhibition. (A–F) HEK293T cells were transfected with ORF9b WT-hemagglutinin (WT-HA) and empty vector VR1012 (VR), HUWE1 WT-Myc, HUWE1 C4341S-Myc mutant, UBR5 WT, or UBR5 C2768A mutant expressing plasmids for 24 h. Then, cells were transfected with RIG-I(N)-Myc for another 8 h. RT-qPCR was conducted to determine the mRNA levels of IFN-β (**A**), IL-29 (**B**), OAS2 (**C**), ISG15 (**D**), ISG56 (**E**), and CXCL10 (**F**). (**G**) Protein samples in Fig. 5A through F were analyzed using IB. (H–J) HEK293T cells were transfected with ORF9b KK4/40RR-HA mutant and empty vector VR1012 (VR), HUWE1 WT-Myc, or UBR5 WT-Myc expressing plasmids for 24 h. Then, cells were transfected with RIG-I(N)-Myc for another 8 h. RT-qPCR was conducted to determine the mRNA levels of ISG15 (**H**), ISG56 (**I**), and CXCL10 (**J**). (**K**) Protein samples in Fig. 5H through J were analyzed using IB. All data are representative of three independent experiments. The data are presented as means ± SD (ns, not significant, **, *P* < 0.01; ***, *P* < 0.001).

ORF9b targets multiple components of the innate immune system to inhibit IFN production and plays an important role in SARS-CoV-2 pathogenesis (26, 27). Here, we examined the effects of HUWE1 and UBR5 on ORF9b-mediated IFN inhibition in HEK293T cells. Reverse transcription-quantitative polymerase chain reaction (RT-qPCR) analysis revealed that ORF9b inhibited the production of IFN-β, interleukin-29 (IL-29), and the four IFN-stimulated genes (ISGs) ISG15, ISG56, 2´-5´-oligoadenylate synthetase 2 (OAS2), and C-X-C motif chemokine ligand 10 (CXCL10) stimulated by RIG-I(N) (the constitutively active form of RIG-I). Overexpression of HUWE1 and UBR5 resulted in weaker IFN inhibition by ORF9b WT (Fig. 5A through G). However, the two HECT domain mutants, HUWE1 C4341S and UBR5 C2768A, had no effect on ORF9b-mediated inhibition of IFN production due to their loss of ability to degrade ORF9b. Similarly, the ORF9b KK4/40RR mutant lacked the ubiquitination sites for HUWE1 and UBR5; the mRNA levels of *ISGs* decreased by ORF9b KK4/40RR mutant were not affected by overexpression of HUWE1 or UBR5 (Fig. 5H through K). Taken together, these results suggest that HUWE1 and UBR5 serve as antiviral factors inhibiting the ORF9b-mediated immune escape.

**Fig 6:**
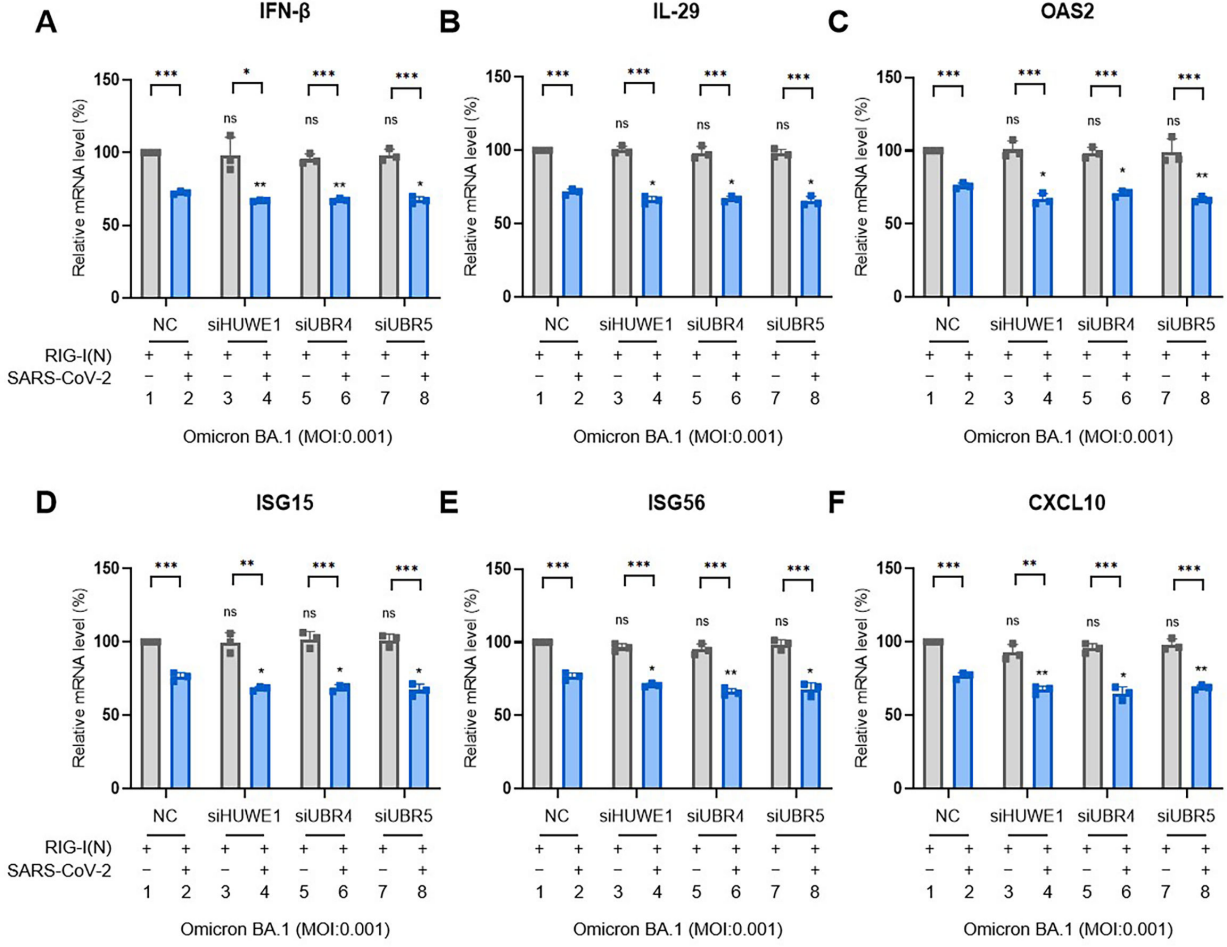
Knockdown of HUWE1, UBR4, or UBR5 further attenuated the mRNA levels of IFNs and ISGs stimulated by RIG-I(N) in cells infected with SARS-CoV-2. (A–F) Caco2 cells transfected with individual E3 ligase siRNA as indicated were transfected with RIG-I(N)-Myc for 8 h with and without Omicron strain infection at 0.001 multiplicity of infection (MOI）for 48 h. RT-qPCR was conducted to determine the mRNA levels of IFN-β (**A**), IL-29 (**B**), OAS2 (**C**), ISG15 (**D**), ISG56 (**E**), and CXCL10 (**F**). All data are representative of three independent experiments. The data are presented as means ± SD [ns, not significant; *, *P* < 0.05; **, *P* < 0.01; ***, *P* < 0.001 compared with negative control (NC)].

To further confirm the relevance of these three E3 ligases with IFN production, we examined the effect of UBR4 or HUWE1 or UBR5 knockdown on the mRNA levels of IFNs and ISGs when Caco2 cells were infected with SARS-CoV-2 (Fig. 6A through F). The results showed that knockdown of any of these three E3 ligases further attenuated IFN and ISG mRNA levels stimulated by RIG-I(N) in cells infected with SARS-CoV-2.

### HUWE1, UBR4, and UBR5 attenuate the replication of SARS-CoV-2

**Fig 7:**
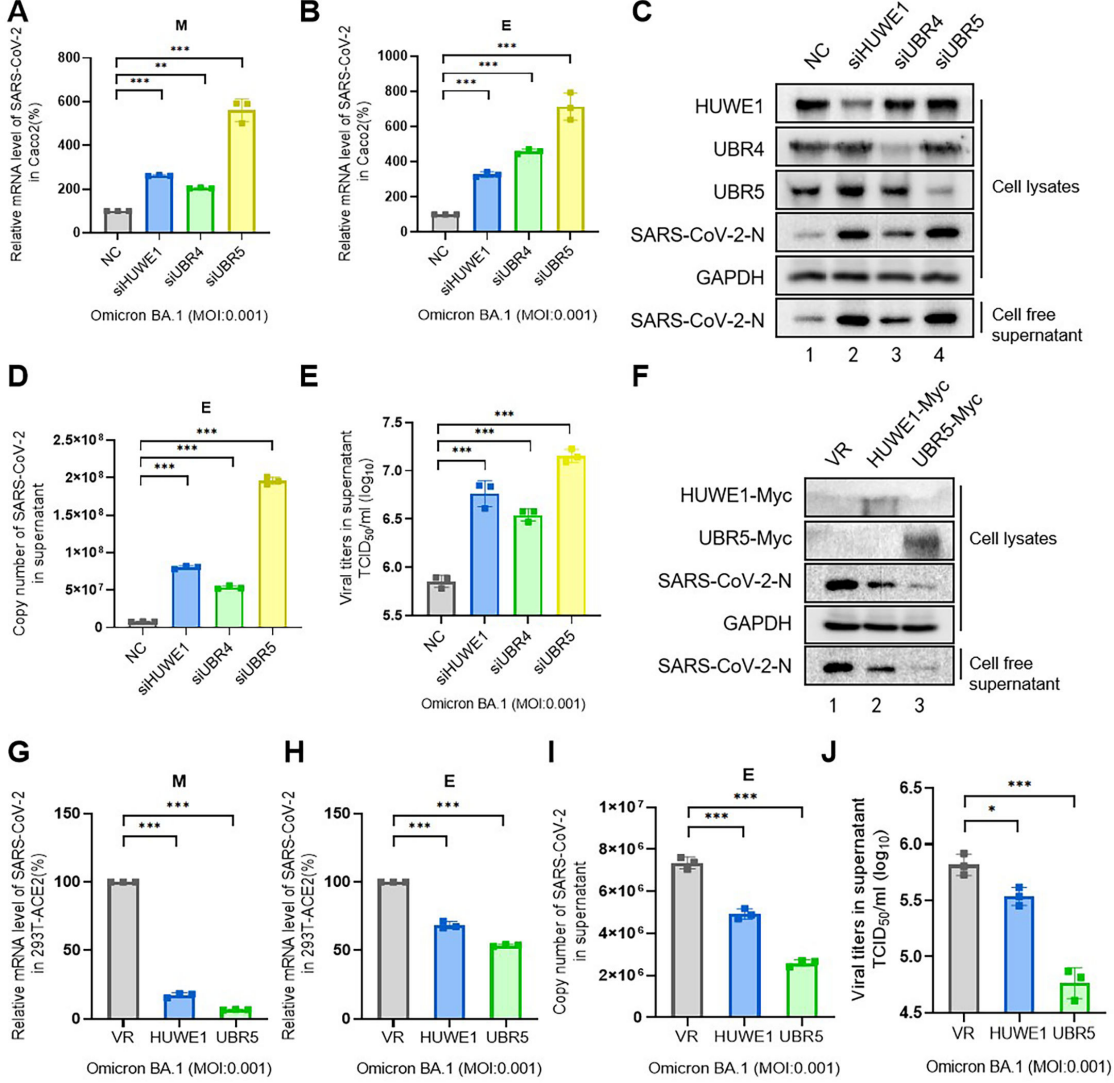
The E3 ligases HUWE1, UBR4, and UBR5 suppressed SARS-CoV-2 viral replication. (A–E) Knockdown of any one of three E3 ligases promoted the replication of SARS-CoV-2 Omicron strain. Caco2 cells transfected with individual E3 ligase siRNA as indicated were infected with Omicron strain at 0.001 MOI for 48 h and harvested. RNAs of SARS-CoV-2 *M* and *E* were detected by RT-qPCR (**A, B, and D**), and the virus titer in supernatant (**E**). The expression of SARS-CoV-2 N of intracellular and cell-free supernatant, HUWE1, UBR4, UBR5, and glyceraldehyde-3-phosphate dehydrogenase (GAPDH) were detected by IB (**C**). (F–J) HEK293T-ACE2 cells that stably expressed HUWE1, UBR5, or empty vector VR1012 were infected with Omicron strain at 0.001 MOI for 48 h, and cells and supernatants were harvested for the further detections. The expression of SARS-CoV-2 N of intracellular and cell-free supernatant, HUWE1, UBR5, and GAPDH were detected by IB (**F**). RNA of SARS-CoV-2 *M* and *E* were detected by RT-qPCR (G–I), and the virus titer in supernatant (**J**). All data are representative of three independent experiments. The data are presented as means ± SD (*, *P* < 0.05; **, *P* < 0.01; ***, *P* < 0.001).

ORF9b is a key innate immune antagonist of SARS-CoV-2 that enhances viral innate immune evasion by reducing or delaying the early host innate responses, and thus facilitates viral replication (6, 7). In this study, we investigated the effects of HUWE1, UBR5, and UBR4 on the replication of SARS-CoV-2. Following HUWE1, UBR4, or UBR5 knockdown, a significant increase in the mRNA levels of intracellular *M* and *E* genes, the protein level of intracellular N using IB, the mRNA level of *E*, and virus titer in the supernatant of Omicron strain was observed, indicating that these three E3 ligases attenuated the viral replication in Caco2 cells (Fig. 7A through E). In contrast, overexpression of HUWE1 and UBR5 decreased the replication of SARS-CoV-2 by detecting intracellular N protein levels of the Omicron strain, the mRNA levels of intracellular *M* and *E* genes and *E* in the supernatant by RT-qPCR, as well as decreased virus titer in the supernatant in HEK293T-ACE2 cells (Fig. 7F through J).

### IFNs promote the mRNA levels of HUWE1, UBR4, and UBR5

**Fig 8:**
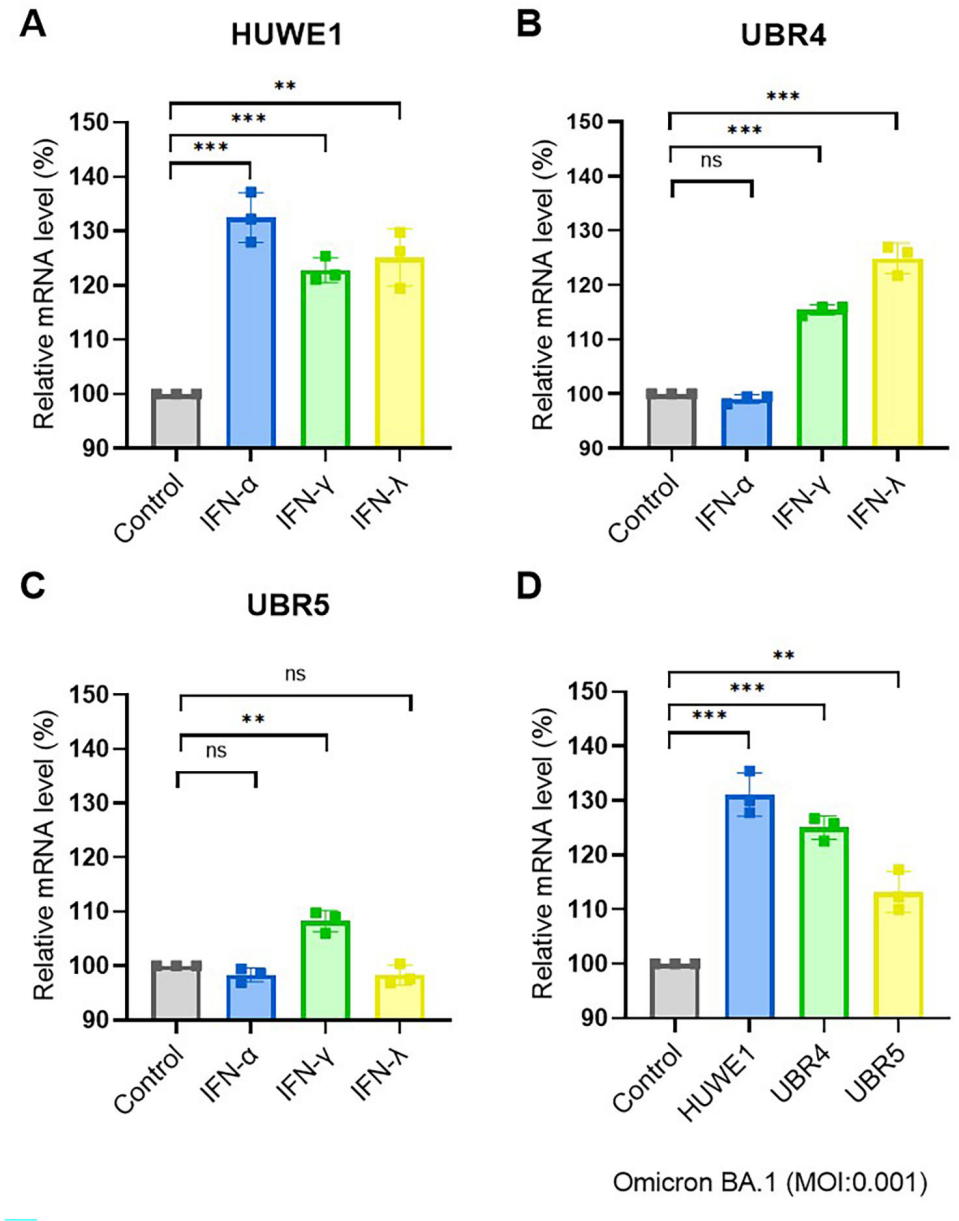
Induction of HUWE1, UBR4, and UBR5 by IFNs. (A to C) Caco2 cells were treated with the indicated IFNs (500 U/mL each) or the vehicle (phosphate-buffered saline) for 8 h, and the mRNA levels of HUWE1, UBR4, and UBR5 were analyzed by RT-qPCR. (D) Caco2 cells were treated with Omicron strain viral infection at 0.001 MOI for 48 h. The mRNA levels of HUWE1, UBR4, and UBR5 were analyzed by RT-qPCR. All data are representative of three independent experiments. The data are presented as means ± SD (ns, not significant; **, *P* < 0.01; ***, *P* < 0.001).

To examine whether HUWE1, UBR4, and UBR5 expression were modulated by IFNs, Caco2 cells were treated with IFNs and the mRNA levels of E3 ligases were quantified using RT-qPCR (Fig. 8A through C). The results showed that IFN-α, IFN-γ, and IFN-λ all upregulated the mRNA level of HUWE1. IFN-γ and IFN-λ upregulated the mRNA level of UBR4, whereas only IFN-γ upregulated the mRNA level of UBR5. Next, the mRNA levels of E3 ligases were assessed again in the context of the virus infection (Fig. 8D). Similarly, the mRNA levels of HUWE1, UBR4, and UBR5 were promoted in Caco2 cells infected with SARS-CoV-2.

## DISCUSSION

Ubiquitination plays an important role in host innate immunity and viral pathogenicity, acting as a double-edged sword for the host and virus (28, 29). Viruses employ viral proteins to hijack the ubiquitination system to target host antiviral factors, including host restriction factors, as well as crucial signaling molecules like MAVS and nuclear factor κB, thereby inhibiting the activation of innate immune responses (30–38). For example, some viral proteins suppress host immunity by deubiquitinating RIG-I and STING or by inhibiting their binding to the relevant E3 ligases to attenuate their activation (39–41). In contrast, the host triggers E3 ubiquitin ligases to degrade viral proteins via UPS, thereby weakening the virulence and pathogenicity of the virus (41–43). Ubiquitination not only leads to protein degradation, but is also involved in other functions, such as mitochondrial translocation, transcriptional enhancement, and maintenance of RNA stability (24). Polyubiquitination may occur at the amino-terminal methionine (M1) or the amine group in one of the seven lysine residues of ubiquitin. For example, E3 ligase HECTD3 promotes RNA virus replication and virus-induced inflammation via K33-linked polyubiquitination of double-stranded RNA-dependent protein kinase (PKR) (44). Here, we found that HUWE1, UBR5, and UBR4 serve as ORF9b E3 ubiquitin ligases and synchronously induce ORF9b K48-linked polyubiquitination and degradation. The study is the first report that SARS-CoV-2 accessory proteins can be independently and simultaneously regulated by multiple E3 ligases (Fig. 1 to 4). In addition, we identified 14 E3 ligases that specifically interact with ORF9b using MS analysis, but only three E3 ligases were relevant to ORF9b degradation. However, the associations between the other 11 E3 ligases and ORF9b, whether they regulate ORF9b functions, and their regulatory mechanisms warrant further investigation.

Although the accessory proteins of coronaviruses are not indispensable for viral replication, some are required for virulence by regulating the host pathways (45, 46). ORF8 protein of SARS-CoV-2 induces endoplasmic reticulum stress-like responses and facilitates viral replication by triggering the calnexin switch, and its genomic deletion attenuates viral replication (47, 48). ORF7a protein of SARS-CoV-2 initiates autophagy and limits the autophagosome-lysosome fusion via the degradation of SNAP29 to promote viral replication (49). A review of the literature shows ORF9b-mediated activation of inflammasome to evade immune responses, facilitating viral replication (1). We therefore speculated that HUWE1, UBR4, and UBR5 may attenuate the replication of SARS-CoV-2, and this was confirmed in Fig. 7.

IFN response is the first line of defense against host immunity. However, patients with coronavirus disease 2019 (COVID-19) exhibit impaired host antiviral responses, including type I/III IFN production and ISG expression (50, 51), owing to various antagonizing mechanisms mediated by multiple viral proteins. SARS-CoV-2 ORF9b is an important IFN antagonist owing to its association with TOM70 and targets multiple components of the RIG-I/MDA-5/MAVS, TLR3-TRIF, and cGAS-STING signaling pathways (4–6, 52). Therefore, the E3 ligases may hinder the ORF9b-mediated IFN inhibition by degrading ORF9b. Here, we identified multiple E3 ligases, including HUWE1, UBR4, and UBR5, that attenuate ORF9b-mediated IFN inhibition (Fig. 5 and 6). Meanwhile, IFNs promoted the mRNA levels of HUWE1, UBR4, and UBR5 (Fig. 8).

Combined with previous studies, we have determined the following critical roles of HUWE1, UBR4, and UBR5 in virus infection. Here, we found that ORF9b was degraded by the proteasome in host cells, and identified HUWE1, UBR4, and UBR5 as its E3 ligases. The IFN signaling pathway was activated upon viral invasion into host cells, and the expression of the three E3 ligases was increased, as confirmed by the data shown in Fig. 8. The three E3 ligases interacted with ORF9b and ubiquitinated it at lysines 4 and 40 via K48-type polyubiquitination, thereby attenuating the anti-IFN function of ORF9b and ultimately inhibiting SARS-CoV-2 immune escape. Moreover, HUWE1, UBR4, and UBR5 exerted an antagonistic effect on SARS-CoV-2 replication. Therefore, HUWE1, UBR4, and UBR5 acted as intracellular anti-SARS-CoV-2 proteins. Our findings regarding the regulation of ORF9b by UPS and the anti-SARS-CoV-2 function of the three E3 ligases provide insights on the SARS-CoV-2 host resistance mechanisms.

Clinical trials have shown that the restoration of types I and III IFNs in COVID‐19 patients is an effective therapeutic option (53). Furthermore, researchers find that HECT family members are overexpressed in primary samples derived from COVID-19-infected patients and COVID-19 mouse models. Importantly, rare germline activating variants in the NEDD4 and WWP1 genes (HECT E3 ligase family members) are associated with severe COVID-19 cases (54). These results reflect the E3 ligases and ORF9b as potential antiviral targets. Our study provides new possibilities for drug development targeting the interaction between the E3 ligases and ORF9b.

Since ORF9b has emerged as a potent IFN antagonist during the early stage of SARS-CoV-2 infection (5), it may overcome E3 ligases antiviral obstacle by counteracting the stimulatory effect of IFNs on E3 ligases. Indeed, during the early phase of SARS-CoV-2 infection, most COVID-19 cases are asymptomatic or exhibit mild to moderate symptoms (55). Therefore, screening of inhibitors targeting ORF9b deserves to be investigated in the future, which might be a great prophylactic agent against COVID-19.

In addition to their roles in SARS-CoV-2, HUWE1, UBR4, and UBR5 have been found to play roles in other viruses. HUWE1 negatively influences HIV-1 infectivity, as a cellular interactor of Gag-Pol through integrase binding (56). UBR5 acts as an antagonist against the Middle East respiratory syndrome coronavirus and diminishes its viral replicative capacity by specifically inducing ORF4b degradation (41). UBR4 is exploited by dengue virus, but not Zika virus, to inhibit IFN-I signaling via STAT2 degradation (20, 57). UBR4 is difficult to manipulate as it contains 106 exons and its encoded protein is approximately 600 kDa. Therefore, we could not successfully construct a UBR4 expression vector in this study, similar to previous studies. So, targeting UBR4 as an antiviral strategy should be further explored in our future studies.

**Fig 9:**
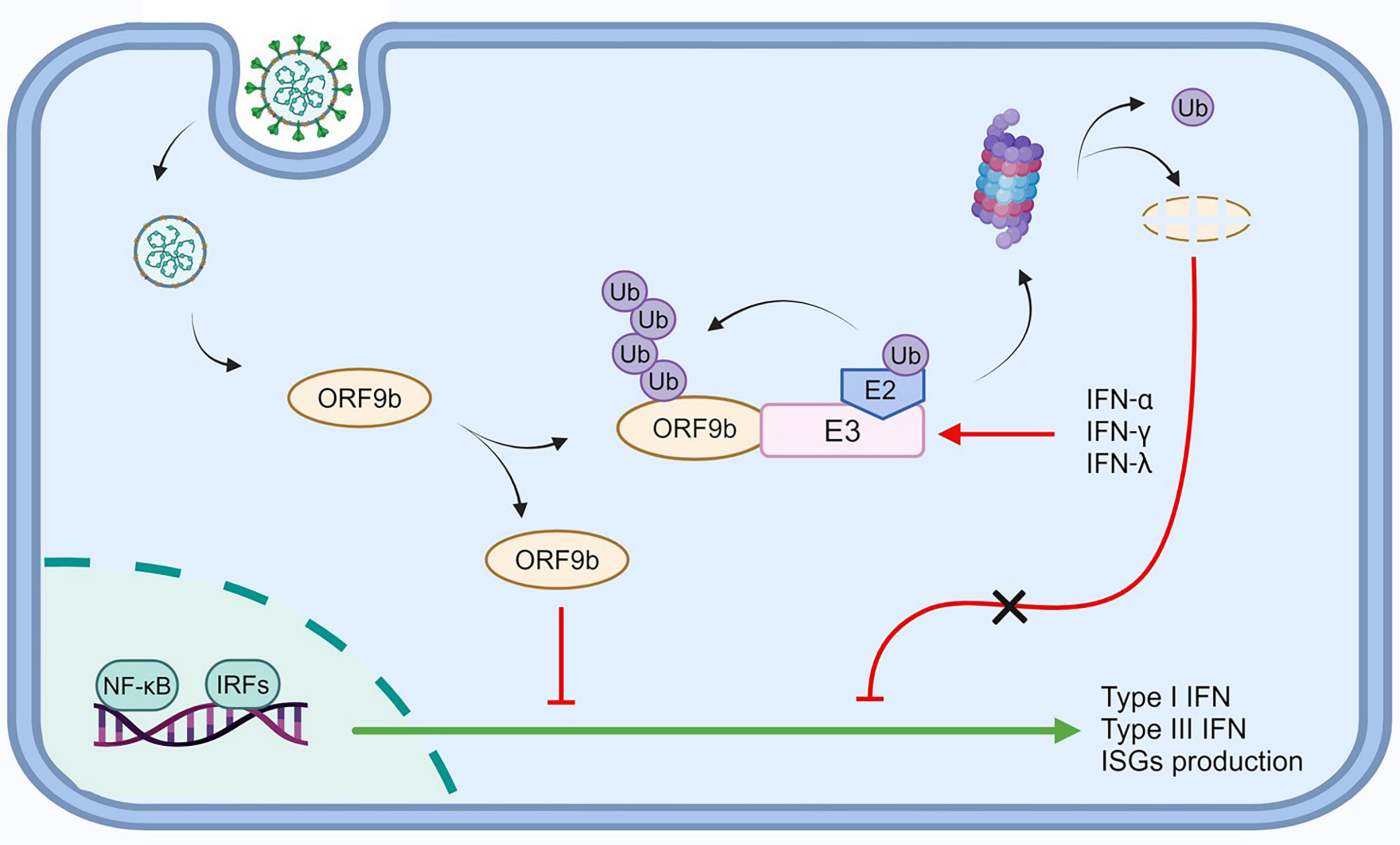
Pattern diagram of HUWE1, UBR4, and UBR5 as antiviral factors against SARS-CoV-2 by promoting degradation of ORF9b (created with BioRender.com.) E3, ubiquitin protein ligase, refers to HUWE1, UBR4, and UBR5; E2, ubiquitin-conjugating enzyme; Ub, ubiquitin; IFN, interferon; NF-κB, nuclear factor kappa B; IRF, interferon regulatory factor; ISG, interferon-stimulated gene.

In conclusion, this study revealed that the HUWE1, UBR4, and UBR5 E3 ligases mediated ORF9b ubiquitination, facilitating its degradation by the proteasome. This proteolytic ubiquitination inhibited the subsequent ORF9b-mediated suppression of type I/III IFN signaling and attenuated the anti-immune ability of ORF9b. Therefore, HUWE1, UBR4, and UBR5 acted as intracellular anti-SARS-CoV-2 proteins that weakened the immune escape of SARS-CoV-2 and exerted an antagonistic effect on SARS-CoV-2 replication (Fig. 9). Our findings provide a basis for the ORF9b-E3 ligase interaction as a novel molecular target, potentially opening new avenues for therapeutic intervention in viral diseases.

## MATERIALS AND METHODS

### Plasmid construction and siRNA oligonucleotides

HUWE1 cDNA (Addgene, catalog no. 37431) and C4341S mutant of HUWE1 (Addgene, catalog no. 37432) were purchased and then inserted into SalI/BglII of VR1012 cytomegalovirus (CMV) promoter (38). UBR5 cDNA (Addgene, catalog no. 81062) and C2768A mutant of UBR5 (Addgene, catalog no. 81065) were individually inserted into VR1012 as SalI/BamHI fragments. ORF9b-hemagglutinin (ORF9b-HA), ORF9b-K4R-HA, ORF9b-K40R-HA, and RIG-I(N)-Myc have been previously described (8). Human ubiquitin protein and its mutants carrying an N terminal Flag tag were inserted into VR1012 as SalI/BamHI fragments. HUWE1-specific siRNA, UBR5-specific siRNA, UBR4-specific siRNA, RLIM-specific siRNA, HECTD1-specific siRNA, TRIP12-specific siRNA, RNF126-specific siRNA, STUB1-specific siRNA, UFL1-specific siRNA, KCMF1-specific siRNA, MYCBP2-specific siRNA, HECTD3-specific siRNA, and WWP2-specific siRNA were synthesized by RIBO Biotechnology Company (Guangzhou, China). A standard scrambled control siRNA was also purchased from RIBO Biotechnology Company. The siRNA sequences are listed in Table 1.

**TABLE 1:** siRNA sequences used in this study

Site	Sequence (5´ to 3´)
Target sequence HUWE1	CAUUGGAAAGUGCGAGUUA
Target sequence UBR5	GCUUCUAAGUUAGAACACA
Target sequence UBR4	CAGAGACAUCCUUUCUCGUACUAAA
Target sequence RLIM	GAUCGAGAAGAAGCUUUCUAUCAAU
Target sequence HECTD1	UGAGCUCGAUGAUGAUGAGGACUUA
Target sequence TRIP12	AGAAUUAGAUUUUCUACUCUC
Target sequence RNF126	CAUCCCGGACGGUACUUCUGCCACU
Target sequence STUB1	AGGCCAAGCACGACAAGUA
Target sequence UFL1	GGAUCCGUCAAGCGAUGAA
Target sequence KCMF1	AGGUGUCAGCUGUGAUGCAUGUUUA
Target sequence MYCBP2	GCACUUUCCUGUGCUUGCCUCUUUA
Target sequence HECTD3	GAGAGGUGCUCUACAAGCUUUACAA
Target sequence WWP2	CCGCAAAGCCCAAGGUGCAUAAUCG

### Cell culture and viruses

HEK293T [American Type Culture Collection (ATCC), Manassas, VA, USA, catalog no. CRL-11268], Caco2 (ATCC, catalog no. HTB-37), and HEK293T-hACE2 (BEI Resources, catalog no. NR-52511) were cultured as monolayers in Dulbecco’s modified Eagle’s medium (DMEM) (Hyclone, Logan, UT, USA) supplemented with 10% heat-inactivated (56°C, 30 min) fetal calf serum (FCS, GIBCO BRL, Grand Island, NY, USA) and maintained at 37°C with 5% CO_2_ in a humidified atmosphere. SARS-CoV-2 Omicron (human/CHN_CVRI-01/2022) viruses were propagated in Caco2 cells and HEK293T-hACE2 cells in DMEM supplemented with 2% FBS, and titered using the median tissue culture infectious dose assay. All experiments of infectious SARS-CoV-2 were conducted under Biosafety Level 3 facilities.

### Transfection and infection

DNA transfections were carried out by Lipofectamine 3000 Reagent (Invitrogen, catalog no. L3000-008) according to the manufacturer’s instruction. siRNA transfections were carried out by Lipofectamine RNAiMAX Reagent (Invitrogen, catalog no. 13778150). For SARS-CoV-2 infection, cells grown to 70% confluence in six-well plates were washed twice with phosphate-buffered saline and incubated with the indicated viral strains at 37°C for 1 h at an MOI of 0.001. Plates were gently agitated at 15-min intervals to facilitate adsorption. After adsorption, virus-containing medium was replaced with fresh medium containing 2% FCS, followed by incubation at 37°C in 5% CO_2_ for the indicated durations.

### Antibodies and immunoblotting

Transfected or infected HEK293T, Caco2, or HEK293T-hACE2 cells were harvested and boiled in 1× loading buffer (0.08 M Tris, pH 6.8, with 2.0% SDS, 10% glycerol, 0.1 M dithiothreitol, and 0.2% bromophenol blue) followed by separation on a 12% polyacrylamide gel. Proteins were transferred onto a polyvinylidene fluoride membrane for IB analysis. The membranes were incubated with primary antibodies, followed by a corresponding horse radish peroxidase (HRP)-conjugated secondary antibody (Jackson Immunoresearch, West Grove, USA, catalog no. 115-035-062 for anti-mouse and 111-035-045 for anti-rabbit) diluted 1:10,000, respectively. Proteins incubated with HRP-conjugated secondary antibody were visualized using the ultra-sensitive ECL chemiluminescence detection kit (Proteintech, Rosemont, IL, USA, catalog no. B500024).

The following antibodies were used in this study: anti-HUWE1 polyclonal antibody (pAb) (Sangon Biotech, Shanghai, China, catalog no. D263109), anti-UBR4 pAb (Sangon Biotech, catalog no. D263223), anti-UBR5 monoclonal antibody (mAb) (Proteintech, catalog no. 66937-1-IG), SARS-CoV-2 nucleocapsid antibody (GeneTex, Irvine, CA, USA, catalog no. GTX635679), anti-Myc pAb, (Proteintech, catalog no. 16286-1-AP), anti-HA pAb (Invitrogen, Carlsbad, USA, catalog no.71-5500), anti-tubulin mAb (Abcam, Cambridge, Cambridgeshire, UK, catalog no. ab11323), anti-Flag mAb (Sigma, Saint Louis, USA, catalog no. F1804), anti-β-actin mAb (GenScript, Nanjing, China, catalog no. A00702), and anti-GAPDH mAb (Sangon Biotech, catalog no. D190090).

### RNA extraction and RT-qPCR

For RT-qPCR, intracellular and viral RNA were extracted from various organizations or cell lines used in this study using TRIzol reagent (Invitrogen), RNase inhibitor (New England BioLabs, Ipswich, MA, USA), and diethyl pyrocarbonate-treated water. The cDNAs were generated by a High-Capacity cDNA Reverse Transcription Kit (Applied Biosystems, Carlsbad, CA, USA) and random or oligo (dT) 18 primers according to the supplier’s instructions. Reverse transcription was carried out in a 20 µL volume, which contained 1 µg RNA extracted from the above samples. RT-qPCR was carried out by the RealMaster Mix (SYBR Green Kit, Takara, Shiga, Japan) and primers designed on an Mx3005P instrument (Agilent Technologies, Stratagene, La Jolla, CA, USA). The RT-qPCR assay was carried out in a 20 µL volume consisting of 1 µL of 5 µmol/L of each oligonucleotide primer, 2 µg of cDNA templates, and 9 µL of 2.5 × RealMaster Mix/20 × SYBR Green solution, which contained the HotMaster Taq DNA Polymerase. Amplifications of the target fragment were carried out as the following steps: initial activation of the HotMaster Taq DNA Polymerase at 95°C for 2 min, and then followed with 40 cycles of 95°C for 15 s, 57°C for 15 s, and 68°C for 20 s. All oligonucleotide primers used for qPCR were listed in Table 2.

**TABLE 2:** Primers used in this study

Primer	Sequence (5´ to 3´)	Purpose
SalI-Myc-HUWE1-F	TGGGTCTTTTCTGCAGTCACCGTCGATGGAACAAAAACTCATATCAGAAGAAGATCTTATGAAAGTAGACAGGACTAAAC	HUWE1-myc-VR1012
HUWE1-BglII-R	TGGCAACTAGAAGGCACAGCAGATCTTAGGCCAGCCCAAACCCTTC	HUWE1-myc-VR1012
SalI-Myc-UBR5-F	TGGGTCTTTTCTGCAGTCACCGTCGACCATGGAACAAAAACTCATCTCTGAAGAGGATCTGATGACGTCCATCCATTTCGTG	UBR5-myc-VR1012
SalI-UBR5-F	TGGGTCTTTTCTGCAGTCACCGTCGACCATGATGACGTCCATCCATTTCGTG	UBR5-VR1012
UBR5-BamHI-R	GCAACTAGAAGGCACAGCAGATCTGCTACACAAAACCAAAATTCTTG	UBR5-myc-VR1012/UBR5-VR1012
M-SARS-CoV-2-RT-F	GGTTTCCTATTCCTTACATGG	Real-time qPCR
M-SARS-CoV-2-RT-R	ATTCTGTAAACAGCAGCAAGC	Real-time qPCR
E-SARS-CoV-2-RT-F	CGATCTCTTGTAGATCTGTTCTC	Real-time qPCR
E-SARS-CoV-2-RT-R	ATATTGCATTGCAGCAGTACGCACA	Real-time qPCR
IFNβ-RT-F	AAACTCATGAGCAGTCTGCA	Real-time qPCR
IFNβ-RT-R	AGGAGATCTTCAGTTTCGGAGG	Real-time qPCR
OAS2-RT-F	AGTCTTAAGAGGCAACTCCG	Real-time qPCR
OAS2-RT-R	AAGGGACTTCTGGATCTCG	Real-time qPCR
ISG15-RT-F	CGCAGATCACCCAGAAGATCG	Real-time qPCR
ISG15-RT-R	TTCGTCGCATTTGTCCACCA	Real-time qPCR
IL-29-RT-F	GAGGCCCCCAAAAAGGAGTC	Real-time qPCR
IL-29-RT-R	AGGTTCCCATCGGCCACATA	Real-time qPCR
ISG56-RT-F	CTAAGCAAAACCCTGCAGAAC	Real-time qPCR
ISG56-RT-R	TCAGGCATTTCATCGTCATC	Real-time qPCR
CXCL10-RT-F	GTGGCATTCAAGGAGTACCTC	Real-time qPCR
CXCL10-RT-R	GACCTTTCCTTGCTAACTGCT	Real-time qPCR
HUWE1-RT-F	TGACCACTGCCAGGAATTTG	Real-time qPCR
HUWE1-RT-R	TGACAGTGTCAATATGGATTTG	Real-time qPCR
UBR4-RT-F	AGTTTGCAGTCTCATTCCCC	Real-time qPCR
UBR4-RT-R	TGTTTCTGGGACACAGCAC	Real-time qPCR
UBR5-RT-F	GCAGTGGTGTAGCACGAAG	Real-time qPCR
UBR5-RT-R	CTCGGTTCCTTAATCTCTGC	Real-time qPCR
GAPDH-RT-F	CCCATCACCATCTTCCAGG	Real-time qPCR
GAPDH-RT-R	TTCTCCATGGTGGTGAAGAC	Real-time qPCR

### co-IP assay

HEK293T cells transfected with the corresponding plasmids were harvested by centrifugation (1,000 *g*, 25°C for 5 min), lysed in lysis buffer (50 mM Tris at pH 7.5, 150 mM NaCl, 1% NP-40, and complete protease inhibitor cocktail tablets) at 4°C for 3 h, and then centrifuged at 12,000 *g* for 10 min. Precleared cell lysates were mixed with antibody-conjugated protein G agarose beads and incubated overnight at 4°C. The next day, the beads were washed six times with washing buffer (20 mM Tris, pH 7.5, 100 mM NaCl, 0.1 mM EDTA, and 0.05% Tween 20) at 4°C, centrifuged at 800 *g* for 1 min. The proteins were eluted with elution buffer (0.1 M glycine-HCl, pH 2.5) and analyzed by SDS-PAGE and immunoblotting.

### MS

HEK293T were transfected with SARS-CoV-2 ORF9b-HA for 36 h, then treated with MG132 or dimethyl sulfoxide (DMSO) for 12 h prior to harvest. Co-IP assays were performed with HA beads (Roche, catalog no. 11867423001), and the elution was analyzed by mass spectrometry. MS analyses were performed by the National Center for Protein Science (Beijing, China).

### Statistical analysis

Experiments were conducted in triplicate, and the data were expressed as the mean ± SD. Data synthesis and analysis were performed using GraphPad Prism software version 8. Statistical comparisons between two groups were made using a Student’s *t*-test. A one-way analysis of variance with Tukey’s multiple comparisons test was used for comparison among the different groups. Significant differences were indicated in figures as follows: ns, no significance; *P < 0.05; **P < 0.01; and ***P < 0.001. *P*-values of less than 0.05 are considered to represent a statistically significant difference.

## Data Availability

The mass spectrometry proteomics data have been deposited to the ProteomeXchange Consortium (https://proteomecentral.proteomexchange.org) via the iProX partner repository with the data set identifier PXD050055.
